# Role of pre-operative endovascular embolization of a giant sacrococcygeal teratoma in neonate: a case report

**DOI:** 10.1186/s43046-024-00216-4

**Published:** 2024-05-13

**Authors:** Isa Azzaki Zainal, Nik Farhan Nik Fuad, Leong Yuh Yang, Nik Azuan Nik Ismail, Nur Yazmin Yaacob, Rozman Zakaria

**Affiliations:** Department of Radiology, Universiti Kebangsaan Malaysia Medical Centre, Kuala Lumpur, Malaysia

**Keywords:** Sacrococcygeal teratoma, Pre-operative embolization, Middle sacral artery, Internal iliac artery, Case report

## Abstract

**Background:**

Giant sacrococcygeal teratomas (SCTs) are at risk of perinatal morbidity and mortality due to their high vascularity. Pre-operative embolization of the feeding arteries, prior to complete surgical resection, may assist in minimizing the intraoperative blood loss by occluding these feeding arteries.

**Case presentation:**

We present a case of a highly vascular giant SCT in a neonate, which was successfully embolized through an endovascular approach prior to surgery. The femoral artery approach was chosen, with access established using a Micropuncture introducer as a sheath. Embolization was performed using a combination of microcoils, Gelfoam slurry, and polyvinyl alcohol particles. The patient developed femoral artery spasm post-procedure, which resolved with the application of a glyceryl trinitrate patch.

**Conclusions:**

Performing pre-operative endovascular embolization on a giant sacrococcygeal teratoma presents particular challenges, primarily due to the difficulty in assessing small vessels and the potential complications associated with this procedure. Nevertheless, this technique proves exceptionally valuable in helping the surgeon minimize blood loss during surgery, thereby reducing the risks of morbidity and mortality. Comprehensive planning for the embolization procedure is essential, encompassing the identification of potential vascular access points and alternatives, along with careful selection of the appropriate catheter.

## Background

Sacrococcygeal teratoma (SCT) is the most common germ cell tumor (GCT) in the neonatal age group, with an incidence of 1 in 30,000 births. Around one-fifth of these neonates also have associated congenital anomalies. It is usually detected during antenatal ultrasound screening [1]. Altman et. al have described four types of SCT: type I is exclusively exterior with a minimal pelvic component; type II has a significant pelvic component, giving rise to an hourglass pattern; type III has larger intra-abdominal and intrapelvic components than the external component; whereas type IV is exclusively presacral with almost no external component [2]. Large hypervascular lesions of more than 10 cm are at risk of perinatal mortality due to uncontrollable bleeding or complications related to congestive cardiac failure [3]. Typically, the main arterial supply of these tumors is a middle sacral artery, with some cases reporting supply from distal branches of the internal iliac artery as well. Definite treatment includes surgical excision (for benign SCTs) and a combination of surgery and chemotherapy (for malignant SCTs). The size and vascularity of the tumor can pose surgical challenges. Pre-operative occlusion of the feeding arteries has proven to be useful in arresting blood supply to the tumors [4].

Based on our literature review of the National Library of Medicine directory, only five cases in English literatures have been published discussing the pre-operative endovascular embolization of the SCT, showcasing various technical approaches to vascular accesses and the use of embolic agents (Table 1).

**Table 1:** Type of SCT and choice of vascular access and embolic agents

Study	Year	Type of SCT	Vascular access	Embolic agents	Others
Cowles et al	2006	Type I	Right femoral artery	Acrylic glue Gelfoam	Radiofrequency ablation
Lahdes-Varma et al	2011	Type II	Left common carotid artery	Gelatin sponges Interlocking coils	N/A
Rossi et al	2013	Type I	Left subclavian artery	Gelatin sponges Microcoils	
Stavropoulou et al	2019	Type II	Left common carotid artery	Microcoils	N/A
Guitart et al	2020	Type I	Left common carotid artery	Gelfoam Microcoils	N/A

Cowles et al. first published a case in 2006 involving a large type I SCT, which underwent both endovascular embolization and percutaneous radiofrequency ablation. They chose right femoral artery as the vascular access point, employing acrylic glue and Gelfoam as embolic agents [5]. In 2011, Lahdes-Vasama et al. reported the successful embolization of a large SCT in a baby using a left common carotid artery as vascular access point, and gelatin sponges and interlocking coils as embolic agents [6]. A similar approach, both in terms of vascular access and embolic agents, was reported in 2020 by Guitart et al.

Meanwhile, Rossi et al. in 2013 presented a case of giant type I SCT that utilized a left subclavian artery approach, employing metallic coils and gelatin sponges as embolic agents [4, 7]. In 2019, Stavropoulou et al. reported a case of type II SCT that underwent endovascular embolization utilizing the left common carotid artery approach and microcoils as embolization agents [8].

## Case presentation

**Fig. 1:**
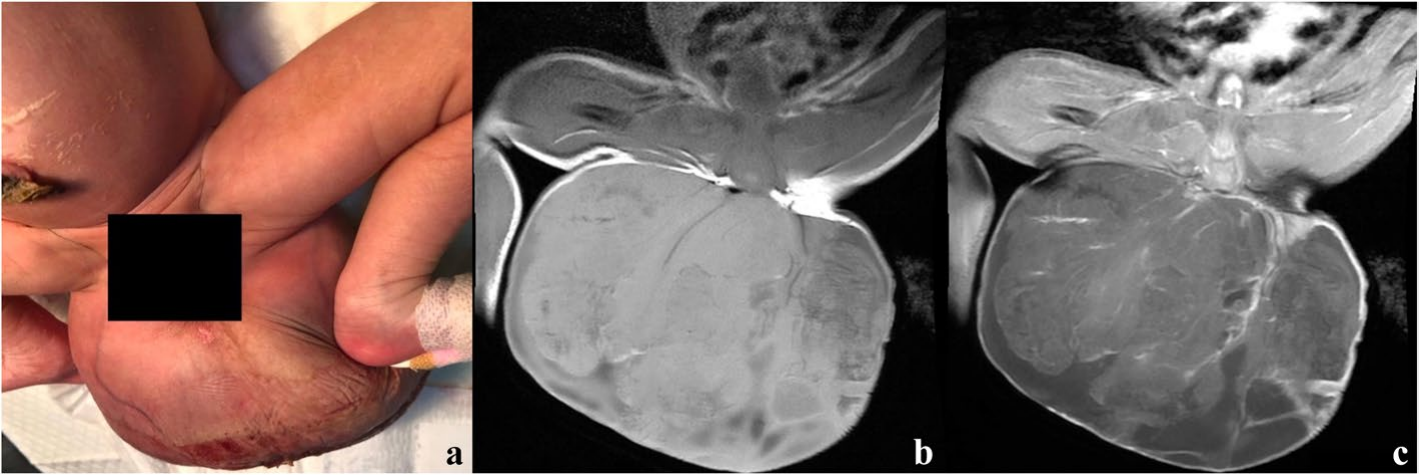
**a** Large sacrococcygeal mass causing abduction of both legs. **b** Unenhanced MRI of the pelvis in coronal T1W view shows large exophytic sacrococcygeal mass with heterogenous signal intensity. **c** Post-gadolinium MRI of the pelvis in coronal T1W view shows minimal enhancement of the exophytic mass

A girl weighing 3.59 kg was born via cesarean section at 38 weeks of gestation, with a large sacrococcygeal mass measuring 15 × 15 cm. Magnetic resonance imaging (MRI) obtained on the third day of life revealed a huge type I SCT with heterogenous signal intensity and minimal enhancement in post-gadolinium images (Fig. 1). The MRI also identified at least three feeding arteries supplying the mass. A few episodes of bleeding from the external surface of the mass were observed and subsequently sutured. In addition, the hemoglobin level dropped from 11.0 to 9.7 g/dL.

**Fig. 2:**
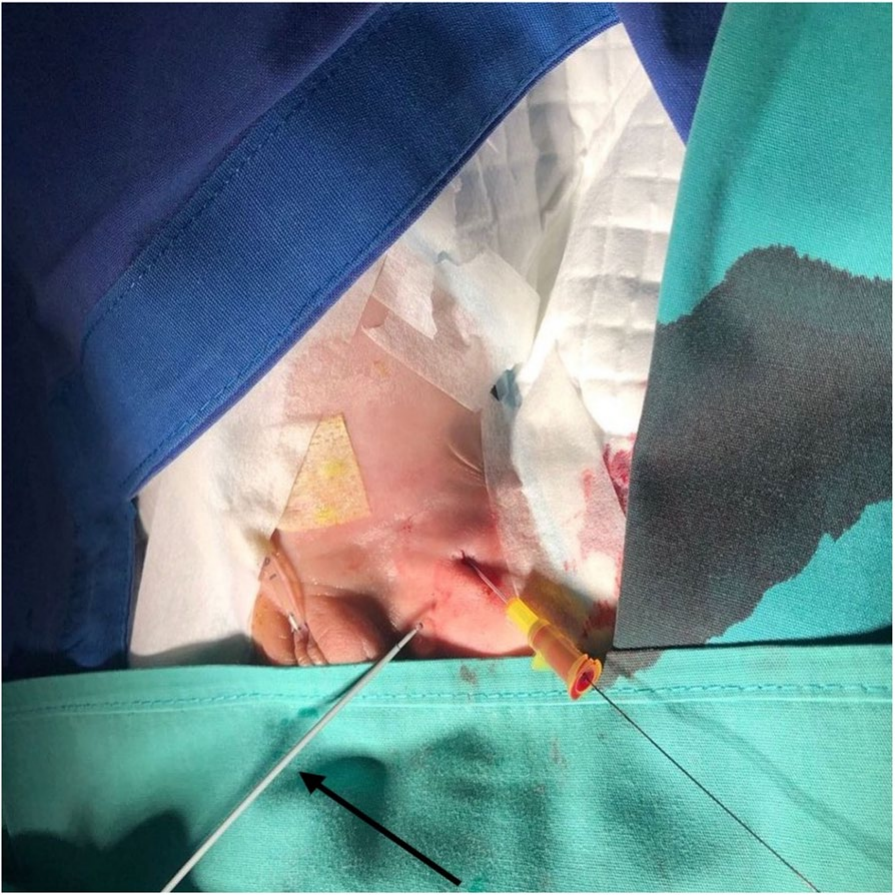
Left femoral artery access using 24G branula, microwire, and Micropuncture introducer (black arrow) as a sheath

As a result, the patient was referred for pre-operative embolization on the seventh day of life, weighing 2.88 kg, indicating a reduction of 20% in weight. The pre-procedural measurement of the left femoral artery was 1.2 mm. While evaluating various vascular access options, the decision was made to proceed with the left femoral artery due to associated higher risk of thrombus formation or stroke with the use of the common carotid artery. The left femoral artery was then punctured using 24G branula under ultrasound guidance. Following this, a 4-Fr Micropuncture introducer was inserted as a sheath, and a 2.7-Fr Progreat microcatheter was used for selective cannulation (Fig. 2). A heparin bolus of 40 U/kg was administered following sheath insertion.

**Fig. 3:**
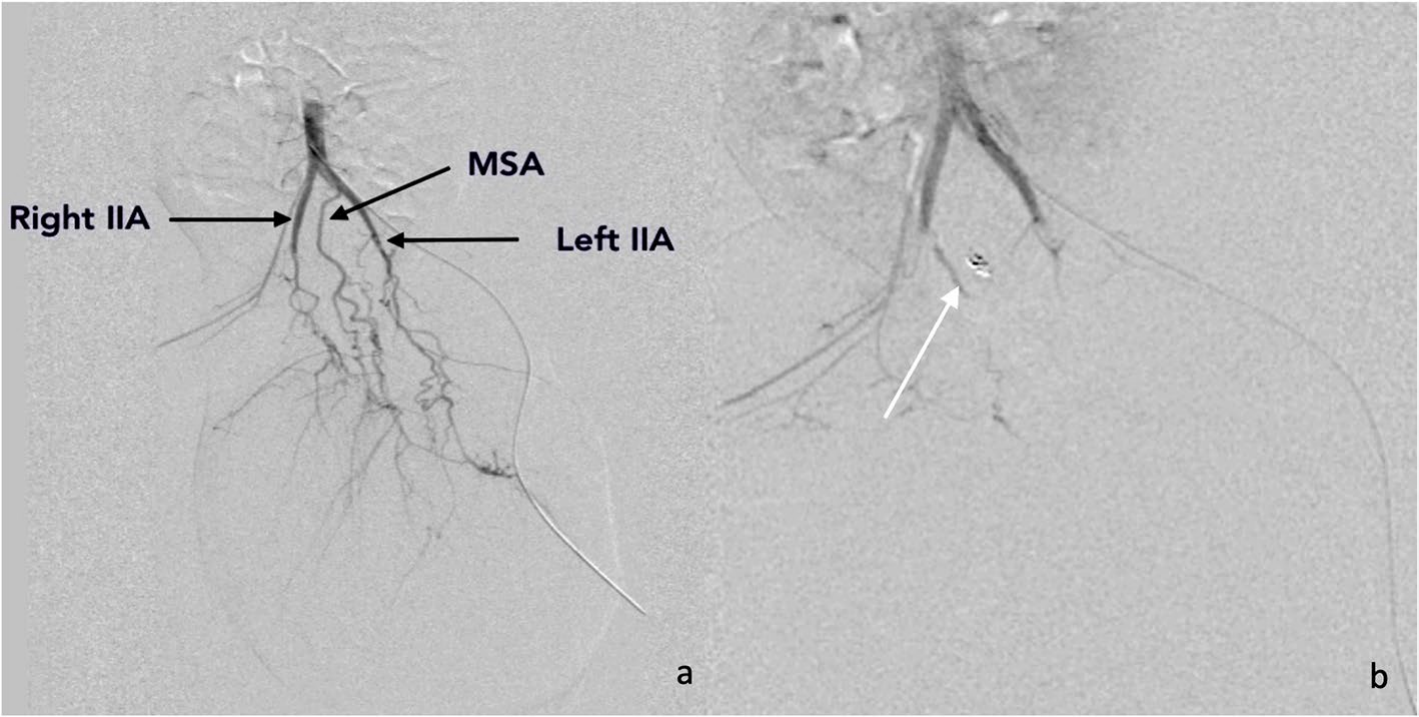
**a** Pre-embolization angiogram shows feeding arteries to the mass. **b** Post-embolization angiogram shows complete occlusion of the feeding vessels. Note coil within the middle sacral artery (white arrow). IIA: internal iliac artery; MSA: middle sacral artery

Angiography confirmed a high-vascular tumor supplied by the middle sacral artery, which was embolized using Contour polyvinyl alcohol (PVA) 150–250 μm and 3 mm × 3 cm coils. The distal branches of bilateral internal iliac arteries were also embolized using Contour PVA 45–150 μm and Gelfoam slurry (Fig. 3a). Post-embolization angiography demonstrated complete occlusion of the feeding vessels (Fig. 3b). A total of 8 ml of non-ionic contrast media was used.

Three hours post-procedure, the patient’s left lower limb exhibited a bluish color, and there was a discrepancy in pulse oximetry reading compared to the contralateral side. Ultrasound assessment revealed a smaller caliber of the left common femoral artery than on the contralateral side. No evidence of thrombus was found; therefore, the patient was treated for post-procedural femoral artery spasm. A glyceryl trinitrate (GTN) patch was applied to the puncture site, and the spasm resolved after 3 days of treatment.

Resection of the tumor was performed 24 h post-embolization, with a total blood loss of 20 ml. Histopathological examination of the excised tumor confirmed it as a sacrococcygeal mature teratoma. The patient was discharged well on the eighth day after surgery, with routine outpatient follow-up.

In this case, we encountered challenges particularly due to the acute anatomical angulation of the femoral artery and feeder arteries. The semi-abduction position of the lower limbs further complicates the femoral artery approach. However, by employing a Micropuncture introducer as a sheath, combined with manipulation of microwire and reshaping of the microcatheter, we managed to achieve adequate catheterization support before proceeding to deliver the embolic materials. For the internal iliac arteries, we opted to use small Contour PVA particles, followed by Gelfoam slurry, to occlude the arteries. Subsequently, the middle sacral artery was embolized using larger Contour PVA particles and a pushable coil.

The small size of the femoral artery further compounded the difficulty, especially with the occurrence of femoral artery spasm following catheterization. However, this was successfully treated with a GTN patch, and no significant disability was observed. Such minor complication could potentially be mitigated by administering a heparin bolus following catheterization or through continuous infusion throughout the procedure.

## Conclusions

In conclusion, the selective pre-operative embolization of the feeding arteries in cases of large hypervascular SCTs is technically safe, feasible, and beneficial in minimizing peri-operative blood loss. Despite the technical and anatomical challenges, the femoral artery approach has proven effective in providing excellent catheterization support for embolization. Furthermore, the combination of embolization materials has yielded outcomes equivalent to those reported in previously documented cases.

## Data Availability

Data sharing is not applicable to this article as no datasets were generated or analyzed during the current study.

## Abbreviations

SCT: Sacrococcygeal teratoma
MRI: Magnetic resonance imaging
PVA: Polyvinyl alcohol
GTN: Glyceryl trinitrate
GCT: Germ cell tumor
